# Glycemic control among patients with type 2 diabetes in a low resource setting in Rwanda: a prospective cohort study

**DOI:** 10.11604/pamj.2022.43.74.35639

**Published:** 2022-10-11

**Authors:** Sadallah Bahizi, Regine Mugeni, Dale Banhart, Chantal Mukankuranga, Gabriel Makiriro, Catherine Kirk, Nesma Lotfy, Maaike Flinkenflogel, Vincent Kalumire Cubaka

**Affiliations:** 1 College of Medicine and Health Sciences, University of Rwanda, Kigali, Rwanda,; 2 Rwamagana Provincial Hospital, Rwanda Ministry of Health, Kigali, Rwanda,; 3 Partners in Health/Inshuti Mu Buzima, Rwinkwavu, Rwanda,; 4 Department of Global Health and Social Medicine, Harvard Medical School, Massachusetts, USA,; 5 University of Global Health Equity, Butaro, Rwanda,; 6 High Institute of Public Health, Alexandria University, Alexandria, Egypt,; 7 KIT Royal Tropical Institute, Amsterdam, Netherlands

**Keywords:** Diabetes, chronic care, primary healthcare, non-communicable diseases, sub-Saharan African

## Abstract

**Introduction:**

diabetes is a leading cause of death, disability, and high healthcare costs, especially among patients with poor glycemic control. Providing decentralized diabetes care to patients in low-income countries remains a major challenge. We aimed to assess hemoglobin A1C (HbA1c) level of patients enrolled in primary-level non-communicable disease clinics of Rwamagana, Rwanda, and identify predictors associated with a) change in HbA1c level over a 6-month period or b) achieving HbA1c <7%. We also explored whether living in a community with a home-based care practitioner was associated with HbA1c-related outcomes.

**Methods:**

we conducted structured interviews and HbA1c testing among patients with type 2 diabetes at baseline and after six months. Multivariable linear regression and multivariable logistic regression were used.

**Results:**

hundred and thirty (130) participants enrolled at baseline, and 123 patients remained in the study after six months. At baseline, 26% of patients had HbA1c <7%. After 6-months, 37% of patients had HbA1c <7%. Factors correlated with the greatest improvements in HbA1c were having HbA1c >9% at baseline, while factors associated with having HbA1c <7% after six months included older age and having HbA1c <7% at baseline. We did not find significant associations between home-based care practitioners and improvement in HbA1c level or achieving HbA1c <7%

**Conclusion:**

the number of patients with well-controlled glycemia improved over time during this study but was still low overall. Care provided by home-based care practitioners was not associated with six-month HbA1c outcomes. Enhanced care is needed to achieve glycemia control in primary healthcare settings.

## Introduction

In 2019, approximately 463 million people were living with diabetes globally, 79% of whom were in low- and middle-income countries [1,2]. Uncontrolled glycemia can lead to serious complications and disability including diabetic retinopathy, nephropathy, neuropathy, foot ulcers, stroke, heart disease, and premature mortality [2-4]. Moreover, costs related to diabetes treatment and management of diabetes-related morbidity and mortality contribute to significant financial burdens on healthcare systems, patients, and their families [2,5-7]. Monitoring glycemia is fundamental to managing diabetes and preventing complications, and glycated hemoglobin (HbA1c) is one of the best ways to monitor how well glycemia has been controlled over the preceding 3-month period [8-11]. Sub-Saharan Africa experiences disproportionate diabetes-related mortality among people aged 20-79 years [2,12,13]. There are tremendous challenges to caring for diabetes patients in sub-Saharan Africa. General healthcare system challenges include a shortage of trained healthcare workers, limited resources, lack of transportation and access to healthcare, and an inconsistent supply chain of medications and diagnostic equipment, and basic medical supplies [14-17]. Integrating care for chronic diseases into primary healthcare services is one approach for better management of diabetes, by monitoring and tracking diabetes patients in a remote area at a reasonable cost while also reducing the risk of hospitalization [18]. Studies have shown improved glycemia control among diabetes patients attending nurses-led clinics in primary health facilities servicing rural areas of South Africa and Cameroon [16,19]. Rwanda has integrated the management of non-communicable diseases (NCDs), including type 2 diabetes into primary healthcare services [20]. However, less is known about the long-term health outcomes of diabetes patients who are receiving follow-up care at health center-based NCD clinics. In a further effort to decentralize care, Rwanda initiated the home-based care practitioner program (HBCP) to increase access to care of NCDs including diabetes at the community level, and the program is not fully expanded in the country [21,22]. In an effort to understand the health outcomes of patients participating in this decentralized care program, we aimed to i) assess HbA1c level among patients with type 2 diabetes enrolled in primary healthcare level NCD clinics of Rwamagana district; ii) identify predictors associated with a change in HbA1c level over a 6-month period or achieving HbA1c <7% after a 6-months period; and iii) explore whether living in a community with HBCP is associated with an improvement in HbA1c level or achieving HbA1c <7% after six months.

## Methods

**Study design and setting:** we conducted a prospective cohort study of patients with type 2 diabetes enrolled in primary healthcare NCD clinics of the Rwamagana District, in Rwanda. The Rwandan health system is composed of three levels, the tertiary level with referral hospitals; the secondary level with provincial and district hospitals, which are the lowest level of facilities with medical doctors; and the primary level, which includes nurse-led health centers and village-based community health workers [23]. Rwamagana district is located in the eastern province of Rwanda with a population of 310,238 people [24]. Healthcare services in Rwamagana district are provided by one provincial hospital which oversees 14 health centers, 9 health posts, 2 private clinics, and 5 private dispensaries [24]. In Rwanda, community-based health insurance, known as *Mutuelle de Santé*, covers the majority of primary healthcare services, including medical care for diabetes [25]. Rwandans in the lowest income bracket (category 1) receive universal healthcare (*Mutuelle de Santé*). Rwandans in the highest income group pay *mutuelle de santé* annual premiums of approximately $7 USD and other income groups pay about $3 USD, as well as co-pays for any services, received [26]. Patients who are newly diagnosed with type 2 diabetes are stabilized at hospitals where they receive treatment. Patients who are referred back to health centers closer to their homes, are managed with oral medications and have monthly clinic visits and monthly blood glucose tests. Patients who require insulin are cared for at the provincial hospital. Patients are also referred to the provincial hospital for HbA1c tests, and screening for eye problems, foot ulcers, or other diabetes-related complications. At the community level, HBCPs provide additional support for patients who have diabetes. Services include at-home blood glucose testing, blood pressure monitoring, and helping patients navigate challenges faced in adhering to medication [21,22]. Home-based care practitioner program are located at the cell level, where each cell is a cluster of about 3-5 villages [21,22]. At the time of data collection, the program was still in its roll-out phase, and so not all cells had a HBCP. In Rwamagana district, 15% of the cells had HBCPs affiliated with three health centers.

**Study population:** we enrolled patients with type 2 diabetes, aged 20 years or older who were being treated with oral diabetes medication in one of the 14 health centers affiliated Rwamagana district hospital. Due to the relatively small number of patients with diabetes receiving treatment at eligible health centers, all patients with type 2 diabetes receiving treatment at one of the 14 health centers were invited to participate. Informed consent was obtained from all participants. Patients who switched to insulin during the study period were transferred to the hospital for clinical care but were still included in data collection during the 6-month follow-up. However, our analysis only included patients who completed both baseline and six-month follow-up surveys and therefore excluded patients who died, moved outside the study area, or were lost to follow-up over the 6-month period.

**Data collection:** baseline data collection occurred between September 2018 and October 2018. Survey questions included age, sex, and history of comorbidities. Weight and height were also measured. We measured HbA1c at point of care (POC) using SD multicare analyzer. Six months from baseline (March-August 2019), we repeated the interviews. Questions were expanded to obtain pertinent demographic information, such as marital status, education, occupation, ubudehe category, address (including sector, cell, and village), as well as health insurance status, medication history, and whether the patients reported receiving healthcare of HBCP. Glycated hemoglobin was measured again. All data were collected on paper forms by two medical doctors and one nurse who are investigators. Data was entered into Epidata (v.2.0.) software.

**Laboratory analysis:** glycated hemoglobin was measured from a capillary blood sample (finger stick sample) at point of care (POC) and analyzed using the SD multicare analyzer (SD Biosensor manufactured in May 2017) which is a biosensor machine that has shown accuracy and precision in testing HbA1c [27]. For values that were reported by the machine as “HI” (high), we assumed an HbA1c level of 15%, which reflected the maximum value that could be measured by the machine.

**Definitions:** body mass index (BMI) was calculated and defined as underweight (BMI <18.5kg/m^2^), normal weight (18.5 kg/m^2^BMI <25 kg/m^2^), overweight (25 kg/m^2^≤ BMI <30 kg/m^2^), and obese (BMI ≥30kg/m^2^) [28]. Our two primary outcomes were: a) a continuous change in HbA1c level, defined as the difference in HbA1c level from baseline and after six months, and b) a binary outcome of having a HbA1c <7% after six months. This threshold for controlled HbA1c is based on a target for adult patients with diabetes because patients with HbA1c <7% are less likely to develop complications of diabetes [29,30]. These two outcomes provide complementary, but not redundant information. The continuous change outcome allowed us to assess whether the patient´s HbA1c levels was improving while assessing whether patients achieved HbA1c <7% allowed us to assess whether patients had achieved the optimal level of glycemia control to reduce the risk of future diabetes complications. Exposure to the HBCP program was assessed in two ways: 1) home-based care practitioner program status using patients´ cell of residence; ii) self-reported interaction with an HBCP.

**Statistical analysis:** to describe our population, we reported frequencies and proportions of variables. A paired t-test was used to compare the change in mean HbA1c level from baseline and after six months. To identify predictors associated with our primary outcomes, we used multivariable linear regression and multivariable logistic regression. For each outcome, we constructed a multivariable model where predictors were identified using a forward selection approach, where all variables with a p-value <0.20 in the univariable model were considered for inclusion in the multivariable model [31]. Although the primary purpose of this study was not to prospectively evaluate the HBCP program, because the timing of the roll-out of HBCP program in our district coincided with the timing of our study, it provided the opportunity to conduct a preliminary assessment of the program. We assessed the association between both definitions of HBCP and change in HbA1c level using a crude linear model and an adjusted linear model that controlled for previously identified predictors as potential confounders. Similarly, we assessed the association between both definitions of HBCP and achieving HBA1c <7% using a crude logistic regression and adjusted logistic regression model that controlled for previously identified predictors as potential confounders. When interpreting these multivariable models, we followed best practices and only interpreted the measure of association for the hypothesized exposure of HBCP rather than for hypothesized confounders [32]. For all analyzes, we defined statistical significance at ɑ <0.05 level. Data analysis was performed using Stata (v 13.0, 2013, Texas, USA).

**Ethical consideration:** ethical approval was obtained from the University of Rwanda, College of Medicine and Health Sciences Institutional Review Board (Review Approval Notice N0 064/CHMS-IRB/2018). Written informed consent was provided in Kinyarwanda, the local language, and was obtained from all participants who were willing to participate. Participants were not paid to participate.

## Results

**General characteristics:** at baseline, a total of 144 patients with type 2 diabetes receiving oral medication and seeking healthcare for diabetes in one of the 14 health centers were invited to participate in the study. Of these, 130 patients (90%) with type 2 diabetes participated in the baseline survey. Five died before the 6-months follow-up visit, 1 moved to another area, and 1 defaulted from the clinical follow-up visit. In total, the study includes data for the 123 patients who remained in the study period. Of the 123 patients who completed the study, 80% were female, were aged 27-93 years with the mean (±SD) of 55.69 ± 11.59, 18% were above 65 years (elderly), 69% were married and 60% were farmers (Table 1). Approximately one-third (33%) lived in communities that had access to HBCP. Comorbidities in addition to diabetes were present in 38% of patients, and 18% had obesity (Table 2). Over the six-month follow-up, 5% changed their oral medication, and 3% initiated insulin therapy.

**Table 1:** demographic characteristics of participants (N=124)

	N (%)
**Gender**	
Male	25(20.33)
Female	98 (79.67)
**Age**	
≤45 years	26(21.14)
46-65 years	75(60.98)
≥66 years	22(17.89)
**Marital status**	
Married	85(69.11)
Single/divorced/widowed	38 (30.89)
**District**	
Rwamagana District	122 (99.19)
Gasabo District	1 (0.81)
**Education**	
No formal education	40 (32.52)
Primary education	62 (50.41)
Secondary or higher education	21 (17.07)
**Occupation**	
No employment	36 (29.27)
Farmer	74 (60.16)
Other type of employment	13 (10.57)
**Health insurance**	
MUSA (*Mutuelle de Santé*)	114 (92.68)
Other than MUSA	9 (7.32)
**Socioeconomic status**	
Ubudehe category 1 (lowest)	9 (7.32)
Ubudehe category (2-4)	114 (92.68)
**Health centers**	
Attended a health center with HBCP	44 (35.77)
Attended a health center without HBCP	79 (64.23)

**Table 2:** clinical characteristics of participants at baseline and 6-months (N=123)

	Baseline N (%) or Mean (SD)	6-months N (%) or Mean (SD)
**Time since diabetes diagnosis**		
≤5 years	78 (63.41)	---
6-10 years	32(26.02)	---
>10 years	13(10.57)	---
**Comorbidities**		
Diabetes only	76 (61.79)	---
Diabetes with other diseases^*a^	47 (38.21)	---
**Level of BMI**		
Underweight (BMI <18.5 kg/m^2^)	10 (8.13)	---
Normal Weight (18.5 kg/m^2^ ≤ BMI <25 kg/m^2^)	53 (43.09)	---
Overweight (25 kg/^2^ ≤ BMI <30 kg/^2^)	38(30.89)	---
Obese (BMI ≥30 kg/^2^ )	22 (17.89)	---
Change of medication regime during study period ^*b^		
Same treatment	---	113 (91.87)
Change of oral medication	---	6 (4.88)
Oral to insulin-treatment	---	4 (3.25)
**Mean HbA1c (SD)**	8.38% (2.09)	7.73% (0.18)
HbA1c level		
<7%	32 (26.02)	46 (37.40)
7%-8%	35 (28.46)	36 (29.27)
8.1%-9%	20 (16.26)	14 (11.38)
9.1%-11%	20 (16.26)	18 (14.63)
>11%	16 (13.01)	9 (7.32)

* a: other diseases included (hypertension, asthma, HIV and gastritis) *b medication regime: patients were on one drug (Metformin or Daonil) or two drugs (a combination of two) with specific dosage; a change of oral medication (but still on oral medication) was a change of dosage or switch to the combination of two drugs if s/he was on one drug

**Glycemic control:** at baseline, 26% had glycemia control (HbA1c <7%), and the proportion of patients with glycemia control (HbA1c <7%) increased to 37% at the end of the study (Table 2). Mean HbA1c was significantly higher at baseline (Mean: 8.38% ± 2.09) than after six months (Mean: 7.73% ± 0.18). This decrease was statistically significant (p = 0.0001).

**Correlates of glycemia control:** in univariable linear regression, only a higher HbA1c level at baseline was statistically significantly associated with a larger improvement in HbA1c level after six months (p < 0.001) (Table 3). In the multivariable model, which also included gender, baseline HbA1c level remained the only significant predictor of change in HbA1c level. Compared to patients with a HbA1c <7% at baseline, those with a baseline HbA1c of 9.1-11% at baseline experienced a -1.59 percentage point decrease in HbA1c on average (95% CI: -2.51, -0.66) while patients with a baseline HbA1c greater than 11% at baseline experienced an average -2.52 percentage point decrease in HbA1c level (95% CI: -3.53, -1.51). In univariable logistic regression, factors that were statistically significant associated with having HbA1c <7% after six months were older age (p = 0.011) and lower HbA1c level at baseline (p < 0.001) (Table 3). In our multivariable model, which included age, ubudehe, and baseline HbA1c, only baseline HbA1c levels remained significant (p < 0.001). Compared to those who had HbA1c <7% at baseline, odds of achieving glycemic control after six months were much lower among participants having baseline HbA1c measurements of 8.1-9% (OR: 0.10, 95% CI: 0.02, 0.45), 9.1%-11% (OR: 0.08, 95% CI: 0.02, 0.36), and above >11% (OR: 0.10 95% CI: 0.02, 0.56). In both our crude and adjusted models, we observed no significant associations between living in a community with HBCP and change in HbA1c level or attaining HbA1c <7% after 6 months (Table 4). In our sensitivity analysis that used self-reported home care with HBCP, we observed an association between self-reported HBCP and a 0.80 increase in HbA1c (95% CI: 0.44-1.56, p= 0.038) in the adjusted model, but no association between self-reported HBCP and having HbA1c <7% after 6-months.

**Table 3:** predictors of change in HbA1c level (assessed using linear regression) and having HbA1C <7% at 6-months (assessed using logistic regression) (N=123)

	Change in HbA1c level		Having HbA1c <7% after 6 months	
	Univariable	Multivariable	Univariable	Multivariable
	β (95% CI)	β (95% CI)	OR (95% CI)	OR (95% CI)
**Gender**	*	*		
Male	-ref-	-ref-	-ref-	
Female	-0.81 (-1.62-0.00)	-0.64 (-1.40-0.13)	0.57 (0.24-1.40)	
**Age**			**	*
≤45 years	-ref-		-ref-	-ref-
46-65 years	0.27 (-0.57-1.10)		5.40 (1.49-19.58)	3.53 (0.88-14.16)
≥66 years	0.62 (-0.44-1.68)		9.20 (2.12-39.89)	5.35 (1.10-26.07)
**Marital status**				
Married	-ref-		-ref-	
Single/divorced/widowed	0.10 (-0.62-0.81)		1.56 (0.72-3.41)	
**Education**				
No formal education	-ref-		-ref-	
Primary education	-0.34 (-1.08- 0.40)		0.74 (0.33-1.68)	
Secondary or higher	0.10 (-0.89-1.09)		0.68 (0.22-2.04)	
**Occupation**				
No employment	-ref-		-ref-	
Farmer	0.36 (-0.39-1.10)		0.96 (0.42-2.17)	
Other	0.74 (-0.45-1.92)		0.70 (0.18-2.71)	
**Health insurance**				
*Mutuelle de Santé*	-ref-		-ref-	
Other	-0.12 (-1.40-1.15)		1.37 (0.35-5.39)	
**Socioeconomic status**			*	*
Ubudehe category (2-4)	-ref-		-ref-	-ref-
Ubudehe category 1	-0.17 (-1.44-1.10)		3.70 (0.88-15.59)	3.24 (0.63-16.63)
**Time since diabetes diagnosis**				
≤5 years	-ref-		-ref-	
6-10 years	0.38 (-0.39-1.15)		0.65 (0.27-1.56)	
>10 years	-0.02 (-1.12-1.08)		0.64 (0.18-2.25)	
**Comorbidities**				
Diabetes only	-ref-		-ref-	
Other comorbidities	0.44 (-0.24-1.11)		0.79 (0.37-1.69)	
**BMI**				
Underweight (BMI <18.5 kg/m^2^)	-ref-		-ref-	
Normal weight (18.5 kg/m^2^ ≤ BMI <25kg/m^2^)	0.19 (-1.07-1.45)		4.63 (0.54-39.45)	
Overweight (25 kg/m^2^ ≤ BMI <30 kg/m^2^)	0.56 (-0.74-1.87)		8.10 (0.93-70.37)	
Obese (BMI ≥30 kg/m^2^)	0.85 (-0.55-2.24)		6.23 (0.67-58.18)	
**HbAc1 level at baseline**	***	***	***	***
<7%	-ref-	-ref-	-ref-	-ref-
7%-8%	-0.43 (-1.22-0.37)	-0.22 (-1.05-0.60)	0.38 (0.14-1.04)	0.45 (0.16-1.27)
8.1%-9%	-0.59 (-1.52-0.33)	-0.39 (-1.34-0.55)	0.08 (0.02-0.34)	0.10 (0.02-0.45)
9.1%-11%	-1.69 (-2.61--0.76)	-1.59 (-2.51--0.66)	0.08 (0.02-0.34)	0.08 (0.02- 0.36)
>11%	-2.70 (-3.69--1.70)	-2.52 (-3.5--1.51)	0.06 (0.01-0.34)	0.10 (0.02-0.56)

* p<0.20, **p<0.05, ***p<0.001

**Table 4:** association between receiving of home-based care provider and HbA1c outcomes (change in HbA1c level and having HbA1c <7% after 6-months) (N=123)

	Change in HbA1c level				Having HbA1c <7% after 6 months			
	Crude		Adjusted^1^		Crude		Adjusted^2^	
	β (95% CI)	p-value	Β(95% CI)	p-value	OR (95% CI)	p-value	OR (95% CI)	p-value
HBCP status using cell of residence	0.63 (-0.06, 1.33)	0.075	0.50 (-0.13, 1.13)	0.119	1.01 (0.46, 2.19)	0.987	0.76 (0.30, 1.94)	0.561
Self-reported interaction with an HBCP	0.71 (-0.14, 1.57)	0.102	0.80 (0.44, 1.56)	0.038	0.95 (0.36, 2.47)	0.912	0.74 (0.23, 2.34)	0.612

**^1^**Adjusted for gender and HbA1c at baseline; **^2^**adjusted for age, ubudehe category, and HbA1c level at baseline

## Discussion

Our study found that glycemic control among type 2 diabetes patients enrolled in outpatient NCD clinics of health centers improved over time. The share of patients with well-controlled glycemia in our study increased from 26% at baseline to 37% after six months in primary-level health facilities in rural Rwanda. While fewer than half the patients were able to achieve the desired HbA1c level, these levels of glycemic control are similar to what has been previously reported in tertiary facilities in similar settings [33]. Three different cross-sectional studies from sub-Saharan Africa showed similar results: 36.9% of type two diabetes patients at Kenyatta National Hospital, Kenya had HbA1c <7%; 35.4% of diabetes patients from the University of Gondar Referral Hospital in Ethiopia had HbA1c <7%; and 37.9% of diabetes patients at the National Hospital of Abuja in Nigeria had HbA1c ≤7% [34-36]. These results stand in contrast to higher rates of controlled glycemic in the USA where approximately 55% of patients with diabetes had HbA1c≤7%, and in Hong Kong where 52% of patients with type 2 diabetes on oral medication had HbA1c ≤7% [33,37]. The high rate of poor glycemic control in sub-Saharan African diabetes patients point to a high need of more quality care, especially in light of the emerging diabetes pandemic [2,13].

Our study also contributes to the body of evidence showing glycemic improvement among patients who receive nurse-led community-based care. Although overall glycemic control remained low after six months, this study demonstrated an improvement in HbA1c levels among patients followed at the health center level. Similarly, a retrospective study from three district hospitals in Rwanda found improved glycemic control among diabetes patients (type 1 and 2) who received follow-up care at nurse-led clinics [13]. Other studies, done in Cameroon and South Africa, also have shown that there is an improvement in patients with diabetes who are followed in nurse-led primary healthcare settings [16,19]. We found that patients with higher levels of HbA1c at baseline experienced greater improvements in HbA1c level but were also less likely to attain HbA1c <7% after 6 months. Greater improvements in HbA1c levels among patients with HbA1c >9% at baseline could reflect that these patients had more room to improve compared to patients with lower HbA1c levels at baseline. It is possible that patients with higher HbA1c levels at baseline were also more motivated to adhere to medication and lifestyle changes or received stronger counseling during NCD clinic visits. However, our results also suggest that patients with high HbA1c levels may require more than six months of treatment and behavior intervention to achieve good glycemic control. It has been shown that it can be much harder for patients with HbA1c >9% to achieve HbA1c <7% than for patients to sustain HbA1c <7% [38]. Studies suggest that to achieve optimal glycemic control, patients require a combination of lifestyle changes, drug therapy, and monitoring glycemia [30]. Similarly, previous research at a tertiary hospital in Rwanda has shown that patients who received lifestyle education were significantly more likely to achieve well-controlled glycemia compared to those who did not [33]. Our study showed that management at health facilities was associated with some improvements in glycemic control, however, our results suggest more comprehensive diabetes management is needed to ensure that patients can reduce and maintain target HbA1c to prevent future diabetes-related complications.

Living in a community with an HBCP was not associated with an improvement in HbA1c level or achieving HbA1c <7%, and, in one analysis, we observed a small association between HBCP and increased HbA1c level. We have not found previous studies conducted on Rwanda´s HBCP model, but previous studies have shown that home-based primary care in high-income countries improves outcomes among elderly patients who have diabetes and hypertension [39]. Although we did not observe the expected association, it is important to interpret these results in the context of the program´s early implementation. The HBCP program was implemented only one year prior to our baseline study. The effectiveness of the program may improve as HBCP providers gain more experience and are better integrated into the healthcare system. In general, the HBCP model could be a valuable program to improve the healthcare of patients with diabetes; however, our study suggests a need for additional research on the implementation of this program and that quality improvement efforts may be warranted. This study had a small sample size, however, it included almost all participants who were receiving diabetes care at health centers of Rwamagana district and were eligible for participation in the study. Variables, including employment status, which could change over time, and duration of diabetes diagnosis, which could have been difficult for patients to remember, were also assessed through patient self-report and could be subject to misreporting. Our study was not designed as a formal, prospective evaluation of the HBCP program, HBCP program was not randomized, the program was relatively new during this study period with no baseline data, and the quality and intensity of implementation could have varied across cells and health centers. Despite these limitations, we feel that these preliminary findings point to the need for a more rigorous study that fully evaluates the program´s implementation and effectiveness. Finally, this study was conducted among patients receiving care at rural health centers and may not be generalizable to all patient populations in Rwanda, however, it is our hope that these findings are useful to providers in similar rural contexts who are seeking to enhance diabetes care.

## Conclusion

Providing decentralized type 2 diabetes care in low-resource settings is a challenge. We found that patients receiving follow-up care at primary-level health facilities in Rwamagana district can improve glycemic control over a six-month period. Those with high HbA1c levels at baseline experienced greater reductions in HbA1c levels over time, but were unlikely to achieve HbA1c <7% after six months. Access to care from HBCPs was not associated with improvements in HbA1c level or achieving HbA1c <7% after six months. Enhanced diabetes care in primary care settings is needed to achieve well-controlled glycemia among type 2 diabetes patients living in Rwanda.

### What is known about this topic

- Receiving decentralized diabetes care at primary-level health facilities has been associated with reduced HbA1c level in South Africa;
- In Rwanda, nurses providing diabetes care at primary level health have good adherence to diabetes care protocols, but health outcomes of patients who are receiving follow-up care in these clinics was unknown.

### What this study adds

- HbA1c levels improved over six months of follow-up, but the number of patients with well-controlled glycemia was still low overall;
- Patients with higher HbA1c level at baseline experienced greater improvements in HbA1c level but were also less likely to attain HbA1c <7% after 6 months;
- Exposure to home-based care practitioners, a new program designed to decentralize diabetes care into the community, was not associated with improvements in HbA1c outcomes.
