# Erratum: Glycemic control among patients with type 2 diabetes in a low resource setting in Rwanda: a prospective cohort study. The Pan African Medical Journal. 2022;43:74

**DOI:** 10.11604/pamj.2023.44.123.39538

**Published:** 2023-03-10

**Authors:** The PanAfrican Medical Journal

**Affiliations:** 1 The PanAfrican Medical Journal, Yaoundé, Cameroon.

**Keywords:** Erratum, diabetes, chronic care, primary healthcare, non-communicable diseases, sub-Saharan African

## Abstract

This erratum modifies the name of the author of the article: Glycemic control among patients with type 2 diabetes in a low resource setting in Rwanda: a prospective cohort study. The Pan African Medical Journal. 2022;43: 74. doi: 10.11604/pamj.2022.43.74.35639. The error affected only the author’s name and an orthographic error in the abstract of the article. *https://www.panafrican-medjournal.com/content/article/43/74/full/* Pan African Medical Journal. 2022;43: 74. doi: 10.11604/pamj.2022.43.74.35639

## Erratum

The name of the author was written Dale Banhart instead of Dale Barnhart in the article “Glycemic control among patients with type 2 diabetes in a low resource setting in Rwanda: a prospective cohort study. The Pan African Medical Journal. 2022;43: 74” [1]. This erratum corrects the name of the author into Dale Barnhart. And also correct this orthographic error in the abstract “After 6 months” instead of “After 6-months”.

## References

[1] Bahizi S, Mugeni R, Barnhart D, Mukankuranga C, Makiriro G, Kirk C (2022). Glycemic control among patients with type 2 diabetes in a low resource setting in Rwanda: a prospective cohort study. The Pan African Medical Journal.

